# Development of the process of determining essential hazardous psychosocial factors of employee stress risk

**DOI:** 10.3389/fpubh.2024.1414695

**Published:** 2024-12-10

**Authors:** Oleg Bazaluk, Vitalii Tsopa, Serhii Cheberiachko, Oleg Deryugin, Olha Nesterova, Svitlana Sokurenko, Vasyl Lozynskyi

**Affiliations:** ^1^Belt and Road Initiative Center for Chinese-European Studies (BRICCES), Guangdong University of Petrochemical Technology, Maoming, China; ^2^Department of Management and Economics, International Institute of Management, Kyiv, Ukraine; ^3^Dnipro University of Technology, Dnipro, Ukraine

**Keywords:** psychosocial risk, stress, social factors, work organization, hazards, health psychosocial risk, health

## Abstract

The purpose of the study is to determine the impact of hazardous psychosocial factors on stress among employees when performing production tasks to develop recommendations for reducing their impact. Based on the recommendations of the ISO 45003:2021 standard, a special questionnaire was developed to determine hazardous psychosocial factors that lead to the appearance of worker’s stress, the answers to which were evaluated on a Likert scale with points from 0 to 4. 68 workers (23 men and 45 women) aged 20 to 45 took part in the survey conducted at industrial enterprises of the Dnipro region in May–June 2023. A questionnaire was developed to identify significant psychosocial hazardous factors in personnel at an industrial enterprise, which includes three groups of psychosocial hazardous factors and consists of thirty questions. A ten-step process for managing significant hazardous psychosocial risks is proposed. It was established that women pay more attention to challenges (psychosocial hazardous factors) that are associated with the organization of work (uncertainty at work, lack of breaks due to workload) and social problems (disrespect, disrespect and inattention to employees, unreasonable behavior towards you by leadership); for men, a significant group of hazardous psychosocial factors is—equipment, working environment, hazardous tasks (fear of performing hazardous work; work is associated with a significant risk to life). The novelty consists in the justification of the process of identifying essential psychosocial factors, which will allow managers to systematically monitor the state of mental health of employees, the psychological climate in the organization and respond in a timely manner to expected problems and develop corrective actions to normalize the situation. The process of managing significant hazardous psychosocial risks has been developed, which consists of ten steps and differs from the known procedure of identifying significant hazardous psychosocial factors on a Likert scale, considering the answers of women and men.

## 1 Introduction

Psychosocial problems among employees, which arise due to excessive workload, mobbing, conflicting demands, employment instability, intimidation, etc. (1) lead to experiencing stress and deterioration of physical health (2). In addition, they affect the psychological climate in the workforce (3) and the results of business in general (4). The latter is associated with strained relations between employees, useless loss of working time, unacceptable professional risk, all together leading to a decrease in labour productivity (5, 6).

According to WHO estimates, due to the decrease in labour productivity, because of stress at work, the losses of the global economy amount to 1 trillion US dollars per year. Every $1 invested in employee mental health has a return of $3 to $5 (7). The effectiveness and efficiency of the enterprise directly depends on the productivity of its employees. And it, in turn, depends on the psychological state of employees and the psychological climate at the enterprise (8).

Therefore, there is a need to improve the psychological state of employees through the introduction of various preventive measures to reduce psychosocial risk at work to an acceptable level (9). Preventive measures include psychosocial support programs for employees (10), cognitive therapy based on awareness (11), improving well-being (12), ensuring a balance between work and rest (13), and others.

At the same time, there is a question about the effectiveness of the proposed preventive measures, which depends on their main components, which are determined based on various studies of psychosocial factors of stress (14). Specialists at Rajagiri College of Social Sciences Center for Mental Health Care emphasize the need for a careful study of the conditions of the production environment to establish a psychological climate in the team based on the relationships between employees to avoid ineffective recommendations. It is also important to have feedback from employees for timely correction of preventive measures based on appropriate assessments of the mental state (15). However, the success of their introduction, for the most part, depends on the good will of the organization’s management (16), which does not allow spreading the developed positive practices to other organizations without a preliminary analysis of the organizational culture, which includes values, moral attitudes and behavior models (17). And this requires the identification of all hazardous psychosocial stress risk factors at the workplace (18, 19).

For the result of the implementation of the specified preventive measures to be effective, it is important to ensure systematic work in the organization (20). For this, you can use the ISO 45003:21 standard “Occupational health and safety management. Psychological health and safety at work. Guidelines for managing psychosocial risks.” The guideline provides relevant recommendations for managing psychosocial risks: policy formation in the management system, leadership institute, communication with employees, planning, operation of various protection programs, performance monitoring and improvement. The application of the ISO 45003:21 standard, despite some shortcomings (21), will be helpful in terms of the development of a psychosocial risk management system if other management systems of the standards of the International Organization for Standardization are already functioning in organizations. For example, ISO 45001:18 or ISO 14001:18. This will allow the integration of similar processes into a single system and reduce the burden due to the reduction of mandatory procedures (22).

Assessing psychosocial risk has certain differences from establishing risks from physical hazards in accordance with ISO 45001:18. This is due to the complex process of establishing psychosocial factors of stress due to the need to involve a considerable number of workers, which introduces a significant part of subjectivity (23, 24). The complexity is also enhanced by the lack of clear cause-and-effect relationships between hazardous psychosocial factors, psychosocial hazard (25). In addition, there is a considerable number of different methods for identifying and assessing hazardous psychosocial factors, which can be grouped into two groups. The first are based on establishing changes in the state of the worker, by measuring changes in their physiological parameters due to the influence of the surrounding production environment (26, 27). The second is based on the employee’s subjective assessment of the impact of the surrounding industrial environment through various questionnaires. The methods of the second group are the most widespread, in particular, the following are often used: Mini Psychosocial Factor (MPF) (28), FPSICO (29), Copenhagen Psychosocial Questionnaire (COPSOQ; COPSOQ II, COPSOQ III) (30), FP-ISR (31) and others. There arises an urgent problem of determining the most appropriate approach.

The problem faced by occupational safety specialists who need to carry out risk assessments at workplaces is to adapt the methods of determining psychosocial risks in accordance with the ISO 45003:2021 standard into the general existing occupational safety and health management system, which is built according to requirements of ISO 45001:2018. At the same time, the risk level is presented as the sum of risks from all identified external and internal hazardous factors associated with a certain hazard (32, 54). To identify all external and internal hazardous factors, SWOT analysis and similar approaches are often used (33), which cannot be applied to the analysis of hazardous psychosocial factors. Therefore, there was a need to develop the process of determining essential hazardous psychosocial factors (aspects of work organization, social factors at work; working environment, equipment, and hazardous tasks), the total impact of which can determine the level of stress risk (34, 35).

The purpose of the study is to develop the process of determining significant hazardous psychosocial risk factors of stress that affect the level of stress risk of employees at work in accordance with the requirements of the ISO 45003:2021:2021 standard.

## 2 Materials and methods

This study involves the development of the process of determining significant hazardous psychosocial factors to further calculate the level of stress risk, which is compatible with the requirements of the ISO 45001:2018 standard. For this, existing methods for determining psychosocial hazards were used on the basis of well-known questionnaires (27, 36), which are proposed to be improved in accordance with the specifics of the organization’s work, the number of employees and the tasks set by the company’s management, which is caused by socio-economic transformations in the company, including social changes [24]. To improve the questionnaires, it is suggested to involve a group of experts who meet certain requirements (work experience, knowledge of the requirements of the standard, knowledge in the field of mental health, and others) (37).

For example, to find out how employees react to frequent changes in the production process, a questionnaire (Table 1) is proposed to determine hazardous psychosocial factors in accordance with the requirements of ISO 45003:2021. It consists of several groups of hazardous psychosocial factors: aspects of organizational work, social factors, equipment, working environment, dangerous tasks were developed for company employees, which are specified in the standard. Each group contains ten questions that were formed based on the level of training of employees, their worldview, awareness of the need for changes, as well as considering examples of already existing similar questionnaires (38). When formulating the questions, attention was paid to the causes of professional stress, possible inadequate attitudes, errors in the performance of tasks, persistent emotional experiences, and the level of motivation (23). In addition, the requirements of national legislation on discrimination, mobbing and sexism were considered.

**Table 1:** Questionnaire form for the identification of significant psychosocial hazardous factors of the personnel at the enterprise.

Designation	Hazardous factor (question)	If: “definitely not”—0; “no more than that”—1; “not no and not so”—2; “yes more than no”—3; “exactly so”—4	
		Answer	Points
1. Aspects of organizational work			
HF_1-1_	Is there uncertainty before the job is done?		
HF_1-2_	Are there production tasks that are difficult to combine?		
HF_1-3_	Do you have to neglect production tasks because of their considerable number?		
HF_1-4_	Do you refuse to take breaks due to busyness?		
HF_1-5_	Do you feel a lack of time to do the work?		
HF_1-6_	Is the amount of work increased for you?		
HF_1-7_	Are there constant demands to complete the work in a tight time frame?		
HF_1-8_	Does multitasking hAPPEN at work?		
HF_1-9_	Is the work schedule inconvenient?		
HF_1-10_	Do you work overtime?		
2. Social factors			
HF_2-1_	Is there lack of support from colleagues or management?		
HF_2-2_	Is the level of interaction between colleagues low?		
HF_2-3_	Does the management lack concern for your well-being?		
HF_2-4_	Is a management style used that does not match the nature of the work?		
HF_2-5_	Does management make unscrupulous decisions?		
HF_2-6_	Have there been incidents between employees involving an overt or covert challenge to health, safety or welfare?		
HF_2-7_	Have you recorded unwanted, offensive, intimidating behavior of colleagues?		
HF_2-8_	Have you recorded ambiguous (more than once) unjustified behavior towards you by the management?		
HF_2-9_	Is there violence at work: threats, assault (physical, verbal or sexual), and gender-based violence?		
HF_2-10_	Is there disrespect and inattention to employees?		
3. Equipment, working environment, hazardous tasks			
HF_3-1_	Is spatial planning of the workplace inappropriate?		
HF_3 = 2_	Is maintenance inadequate?		
HF_3-3_	Are you using outdated equipment?		
HF_3-4_	Is there fear when performing hazardous work?		
HF_3-5_	Does the work involve a significant risk to life?		
HF_3-6_	Are the weather conditions at the workplace unfavourable?		
HF_3-7_	Does the work you perform relate to unstable environments?		
HF_3-8_	Do you perform high-risk/extreme conditions or situations?		
HF_3-9_	Are the necessary workplace safety tools missing?		
HF_3-10_	Are working conditions with technical obligation psychologically exhausting?		

The answers to the mentioned questions are suggested to be evaluated on a 5-point Likert scale from 0 to 4 with a typical answer format: 0–completely disagree, 1–disagree, 2—yes, it happened, 3—agree, 4—completely agree (39–41). The higher the total score, the greater the probability that a person will be under stress of such a level that it can lead to changes in his mental state or the manifestation of psychosomatic diseases (42).

The validity of the determined results based on the use of questionnaires is ensured by the correlation between the statements of employees at similar workplaces and the presence of health disorders among employees (23). This allows making probabilistic statements about the risk of health deterioration based on specific scores on questionnaire scales (23).

When conducting calculations, it was assumed that the presence of an average score for groups of hazardous factors from 2.6 to 4 (Table 2) indicates a significant impact of stress (43), which is characterized by an increase in the general resistance of the body, anxiety, possible nervous disorders, there is a need for more detailed medical examination. If the average score is in the range from 1.6 to 2.5, then we consider that the initial stage of stress is taking place, there are certain signs: anxiety, irritation, indecision, doubts, etc. (44), which indicates the need for increased monitoring of the employee’s condition in order to prevent the development of diseases and the stage of exhaustion of the body’s energy reserves. If the average score is less than 1.5, then we consider that the employee is relatively safe from the influence of psychosocial factors. However, there is still a need to analyse the answers to find out the presence of psychosocial risk factors with a high score.

**Table 2:** Criteria for determining a significant hazardous factor.

№	Criterion	Average score	Influence on the probability and severity of the consequences of experiencing stress	Preventive and protective actions against a hazardous psychosocial factor
1.	Insignificant	0–1.5	Practically absent	No action (possible improvement actions)
2.	Insignificant with verification	1.6–2.5	Moderate	Carry out verification actions—audit on these factors
3.	Significant	2.6–4.0	Critical	Implement preventive and protective measures

The process of determining significant hazardous psychosocial stress risk factors consists of ten steps:

Step 1. We identify hazardous psychosocial factors by group: aspects of organizational work, social factors; equipment, working environment, hazardous tasks. We develop a questionnaire to identify significant psychosocial hazardous factors for the company’s personnel. For example, the given questionnaire Table 1, which was compiled to identify significant hazardous psychosocial factors from three groups: aspects of organizational work, social factors, equipment, working environment, hazardous tasks.

Step 2. We conduct a survey of participants, offering them the questionnaire Table 1.

Step 3. We calculate the average score on a Likert scale, considering the answers of women and men; also, for a more detailed analysis, it is recommended to highlight a group of managers and groups of workers by profession when analysing the survey (we use methods of statistical analysis).

Step 4. Determine significant hazardous psychosocial factors based on the evaluation criteria listed in Table 2.

Step 5. For non-significant hazardous factors, we conduct a periodic, for example, once every six months, determination and analysis of significant hazardous psychosocial factors and response to them.

Step 6. An assessment of the risk of stress is carried out based on the identified significant psychosocial hazardous factors, followed by the development of preventive measures to reduce the risk of stress on employees.

Step 7. Psychosocial stress risks are being documented.

Step 8. A register of significant hazardous psychosocial factors is being created.

Step 9. A plan of measures to reduce preventive measures the significance of hazardous psychosocial factors is being developed.

Step 10. Periodic review of the register of significant hazardous psychosocial factors is ensured.

**Figure 1:**
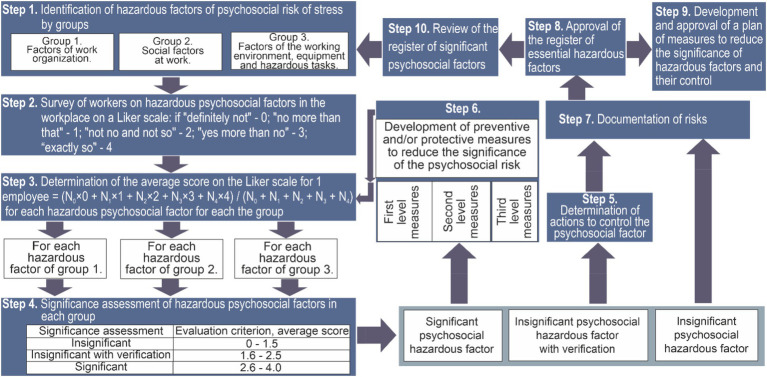
The process of managing significant hazardous psychosocial stress risks.

The process of managing significant hazardous psychosocial risks is clearly shown on Figure 1.

To give an example, a corresponding survey was conducted at industrial enterprises of the Dnipro region among workers of diverse types of industrial activity in the age group from 20 to 45 years old to identify the most significant psychosocial hazardous factors that increase a person’s experience of stress. In total, 68 participants (23 male and 45 female) took part in the study. The research was conducted in May–June 2023. Most of the participants (70%) were married and had at least one child. At the same time, the average length of service at one place was 11.4 years. Also, most participants reported that they work at least 40 h a week. At the same time, their professional activities are related to construction (26.8%), the service sector (31.4%), agriculture (4.2%), and self-employment (14.6%).

Score calculations and determination of measurement discrepancies were performed in Microsoft Excel 2016. Outliers were tested using Z-scores, and all values were within *p* < 0.05. Initial correlations and analyses of variance were conducted to assess covariance and associations between demographic variables (i.e., age, gender, type of industry).

## 3 Results

An example of the formation of the answers of the research participants (female) is given in Table 3, from which the average result is formed for each hazardous factor, which allows identifying significant ones among them. Tables 4–6 show the results of a survey of research participants regarding the impact of psychosocial hazardous factors at the enterprise during the performance of production tasks according to three groups of hazardous factors.

**Table 3:** Report on the survey of all personnel (without division into female and male) on the identification of significant psychosocial hazardous factors among the personnel at the enterprise.

Designation of dangerous factors	The number of employees—N_m_ answered points, respectively,					Average score	Assessment of significance
	0	1	2	3	4		
1. Aspects of organizational work							
HF_1-1_	4	7	15	14	5	2.8 ± 0.3	Significant
HF_1-2_	3	8	16	14	4	2.7 ± 0.4	Significant
HF_1-3_	2	10	17	12	4	2.5 ± 0.2	Insignificant with verification
HF_1-4_	1	9	15	15	5	2.8 ± 0.4	Significant
HF_1-5_	1	8	17	16	3	2.8 ± 0.4	Significant
HF_1-6_	2	6	15	18	4	3.1 ± 0.3	Significant
HF_1-7_	2	10	17	12	4	2.5 ± 0.5	Insignificant with verification
HF_1-8_	3	8	15	15	4	2.6 ± 0.9	Significant
HF_1-9_	5	12	14	12	2	2.1 ± 0.7	Insignificant with verification
HF_1-10_	4	10	16	12	3	2.4 ± 0.4	Insignificant with verification
2. Social factors							
HF_2-1_	5	12	14	12	2	2.1 ± 0.3	Insignificant with verification
HF_2-2_	6	12	14	12	1	1.9 ± 0.3	Insignificant with verification
HF_2-3_	3	10	17	13	2	2.5 ± 0.4	Insignificant with verification
HF_2-4_	6	12	14	11	2	1.8 ± 0.5	Insignificant with verification
HF_2-5_	6	11	15	11	2	1.8 ± 0.5	Insignificant with verification
HF_2-6_	3	10	16	14	2	2.1 ± 0.6	Insignificant with verification
HF_2-7_	2	15	17	10	1	2.3 ± 0.3	Insignificant with verification
HF_2-8_	3	8	14	16	4	2.8 ± 0.3	Significant
HF_2-9_	2	7	16	16	4	2.8 ± 0.3	Significant
HF_2-10_	1	7	16	16	5	2.9 ± 0.3	Significant
3. Equipment, working environment, hazardous tasks							
HF_3-1_	5	12	14	12	2	2.4 ± 0.2	Insignificant with verification
HF_3 = 2_	6	13	14	11	1	1.6 ± 0.3	Insignificant with verification
HF_3-3_	5	10	15	13	2	2.6 ± 0.4	Significant
HF_3-4_	3	7	16	15	4	3.2 ± 0.3	Significant
HF_3-5_	5	11	15	13	2	2.1 ± 0.3	Insignificant with verification
HF_3-6_	6	13	15	10	1	1.6 ± 0.4	Insignificant with verification
HF_3-7_	5	14	15	10	1	1.7 ± 0.5	Insignificant with verification
HF_3-8_	7	12	16	8	2	1.3 ± 0.4	Insignificant with verification
HF_3-9_	6	12	16	10	1	2.3 ± 0.2	Insignificant with verification
HF_3-10_	1	7	16	16	5	1.8 ± 0.3	Insignificant with verification

**Table 4:** The results of a survey of research participants regarding the influence of hazardous psychosocial factors during the performance of production tasks by the group “aspects of organizational work”.

Designation HF_j-i_	Average score for 1 employee = (N_0_ × 0 + N_1_ × 1 + N_2_ × 2 + N_3_ × 3 + N_4_ × 4)/(N_0_ + N_1_ + N_2_ + N_3_ + N_4_)		
	Gender		Total
	Female	Male	
HF_1-1_	2.8 ± 0.4	2.4 ± 0.2	2.6 ± 0.3
HF_1-2_	2.7 ± 0.4	2.5 ± 0.3	2.6 ± 0.3
HF_1-3_	2.5 ± 0.3	2.4 ± 0.3	2.4 ± 0.4
HF_1-4_	2.8 ± 0.4	2.6 ± 0.4	2.8 ± 0.5
HF_1-5_	2.8 ± 0.3	2.6 ± 0.5	2.7 ± 0.7
HF_1-6_	3.1 ± 0.1	2.8 ± 0.5	2.9 ± 0.4
HF_1-7_	2.5 ± 0.2	2.4 ± 0.3	2.4 ± 0.5
HF_1-8_	2.6 ± 0.3	2.4 ± 0.1	2.5 ± 0.6
HF_1-9_	2.1 ± 0.3	2.3 ± 0.2	2.2 ± 0.4
HF_1-10_	2.4 ± 0.2	2.2 ± 0.2	2.3 ± 0.3
The average score for the first group of factors x`	2.63 ± 0.21	2.46 ± 0.13	2.54 ± 0.18

**Table 5:** The results of a survey of research participants regarding the influence of hazardous psychosocial factors during the performance of production tasks by the “social factors” group.

Designation HF_j-i_	Average score for 1 employee (N_0_ × 0 + N_1_ × 1 + N_2_ × 2 + N_3_ × 3 + N_4_ × 4)/(N_0_ + N_1_ + N_2_ + N_3_ + N_4_)		
	Gender		Total
	Female	Male	
HF_2-1_	2.1 ± 0.1	1.9 ± 0.2	2 ± 0.9
HF_2-2_	1.9 ± 0.2	1.8 ± 0.3	1.85 ± 0.2
HF_2-3_	2.5 ± 0.2	2.6 ± 0.2	2.55 ± 0.2
HF_2-4_	1.8 ± 0.3	1.9 ± 0.2	1.85 ± 0.1
HF_2-5_	1.8 ± 0.1	2.1 ± 0.4	1.95 ± 0.5
HF_2-6_	2.1 ± 0.2	2.2 ± 0.5	2.15 ± 0.5
HF_2-7_	2.3 ± 0.3	1.8 ± 0.1	2.05 ± 0.3
HF_2-8_	2.8 ± 0.2	2.2 ± 0.1	2.5 ± 0.2
HF_2-9_	2.8 ± 0.2	2.6 ± 0.2	2.7 ± 0.2
HF_2-10_	2.9 ± 0.2	2.2 ± 0.3	2.55 ± 0.2
The average score for the second group of factors	2.30 ± 0.36	2.13 ± 0.23	2.22 ± 0.28

**Table 6:** The results of a survey of research participants regarding the influence of hazardous psychosocial factors during the performance of production tasks by the group “equipment, working environment, hazardous tasks”.

Designation HF_j-i_	Average score for 1 employee (N_0_ × 0 + N_1_ × 1 + N_2_ × 2 + N_3_ × 3 + N_4_ × 4)/(N_0_ + N_1_ + N_2_ + N_3_ + N_4_)		
	Gender		Total
	Female	Male	
HF_3-1_	2.4 ± 0.1	2.6 ± 0.2	2.25 ± 0.23
HF_3 = 2_	1.6 ± 0.3	1.8 ± 0.2	1.7 ± 0.2
HF_3-3_	2.6 ± 0.2	2.1 ± 0.3	2.35 ± 0.34
HF_3-4_	3.2 ± 0.3	3.4 ± 0.1	3.3 ± 0.4
HF_3-5_	2.1 ± 0.3	2.6 ± 0.1	2.25 ± 0.24
HF_3-6_	1.6 ± 0.2	1.4 ± 0.2	1.5 ± 0.11
HF_3-7_	1.7 ± 0.1	1.6 ± 0.3	1.65 ± 0.24
HF_3-8_	1.3 ± 0.1	1.3 ± 0.4	1.3 ± 0.36
HF_3-9_	2.3 ± 0.2	1.5 ± 0.2	1.85 ± 0.19
HF_3-10_	1.8 ± 0.2	2.9 ± 0.3	2.3 ± 0.3
The average score for the third group of factors	2.06 ± 0.46	2.12 ± 0.60	2.04 ± 0.44

**Figure 2:**
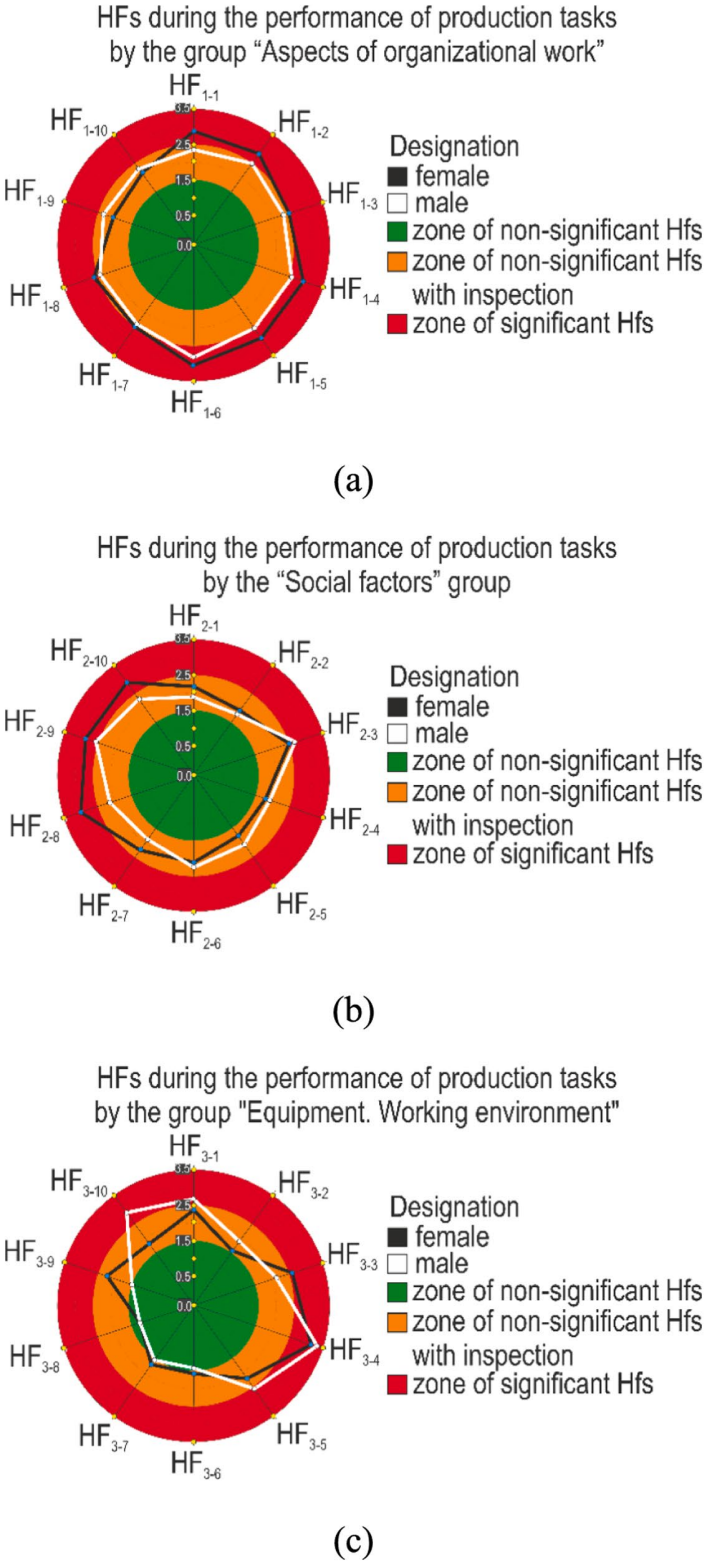
Diagram of assessment of hazardous psychosocial stress risk factors: **(A)** of the “Aspects of organizational work” group; **(B)** “Social factors” group; **(C)** group “equipment, working environment, hazardous tasks”.

The analysis of the obtained results shows (Table 7) that the interviewed females pay more attention to the challenges associated with the organization of work and social factors: the average score is 2.6 and 2.3, while for males it is 2.4 and 2.1, respectively. At the same time, for males, a more significant group of hazardous factors is the group of equipment, working environment, hazardous tasks, and the average score of which was 2.2, while for females it was 2.0. The next important result is the determination of the most influential hazardous factors from each group, the average score of which is greater than 2.5. In this case, it is necessary to provide preventive measures to reduce them. It was established that such hazardous factors for females are HF_1-1,_ HF_1-2,_ HF_1-4_, HF_1-5,_ HF_1-6,_ HF_1-8_ from the factors of aspects of work organization. For males, the number of hazardous factors is slightly less HF_1-4_, HF_1-5,_ HF_1-6_ (Figure 2).

**Table 7:** Comparison of psychosocial hazardous factors of stress risk at the enterprise during the performance of production tasks.

Gender		Total
Female	Male	
Group 1. Aspects of organizational work		
HF_1-1,_ HF_1-2,_ HF_1-4_, HF_1-5_ HF_1-6,_ HF_1-8_	HF_1-4_, HF_1-5_ HF_1-6._	HF_1-1,_ HF_1-2,_ HF_1-4_, HF_1-5_, HF_1-6,_
Group 2. Social factors		
HF_2-8_, HF_2-9_, HF_2-10_	HF_2-3_, HF_2-9_	HF_2-3_, HF_2-10_
Group 3. Social factors		
HF_3-3_, HF3_−4_	HF_3-1_, HF_3-4_, HF_3-5_, HF_3-10,_	HF_3-1_

From this, we can conclude about the significance of three hazardous factors that occur in both groups and require appropriate intervention—the development of preventive measures aimed at revising the number of production tasks, increasing breaks, and approving the appropriate amount of the volume of completed tasks per shift.

In the group of social hazardous factors, we distinguish for female: *HF_2-8_*, *HF_2-9_, HF_2-10_*, and while for males, hazardous factors under the numbers: *HF_2-3_, HF_2-9_.* are important. For females, the issues of ambiguous behavior, the presence of harassment, disrespect and inattention from colleagues are the most worrying, while males are additionally faced with issues related to well-being.

In the group of equipment, working environment, hazardous tasks, females attributed *HF_3-3_, HF3_−4_,* to the most significant factors, while males complained about *HF_3-1_, HF_3-4_, HF_3-5_, HF_3-10_*, which requires the development of preventive measures for updating production equipment, carrying out appropriate professional training for performing work with a high level of injury and hazardous work, as well as psychological preparation for their performance.

The result obtained for all interviewed participants is interesting, which indicates a significantly lower number of significant hazardous factors that occur during the performance of production tasks. This result emphasizes the need to conduct an analysis not in general for all employees, in most cases an acceptable result will be obtained, but to break it down into groups: male, female, by age, by profession, by position, etc., which will allow identifying significant psychosocial factors that need to be dealt with in the future take precautionary measures.

When analysing the results of the presence of psychosocial factors at the workplace, an increase in the indicators of aspects of organizational work and social factors at work is observed. The reason for this may be the deterioration of the cognitive abilities of employees, or the appearance of psychosocial risks at workplaces because of a change in the head of a structural unit, for example. As for the factors of relationships between colleagues/supervisors, this group showed an increase in the percentage of employees who refrained from answering. What can be said about the likely deterioration of relations between employees/management, about which the employee does not want to talk. To identify such facts, it is necessary to conduct additional research in the form of direct communication with employees or a pulse survey on the topic of relationships in the team.

## 4 Discussion

In this study, an attempt was made to develop the process of determining significant hazardous psychosocial risk factors of stress that affect the health of employees when performing production tasks in accordance with the recommendations of ISO 45003:2021. The difference of the proposed approach is the determination of significant hazardous stress risk factors based on a questionnaire developed for a specific enterprise and developed criteria for the significance of a hazardous psychosocial stress risk factor using a Likert scale. This will allow occupational health and safety specialists to apply one of the approaches described in the ISO 31010:2022 standard to determine the risk of stress, and to integrate the psychosocial risk management system into the overall management system of the organization. In particular, having identified significant hazardous psychosocial risk factors for stress, you can further use the appropriate procedure for managing professional risks in the organization according to the ISO 45001:2018 standard.

A feature of the proposed process for determining significant psychosocial risks is the introduction of appropriate scales to identify significant dangerous psychosocial stress risk factors, which can be worked out by involving experts based on the conditions of the production environment and organizational culture (45, 46). Using the Likert scale, it is possible to identify significant hazardous psychosocial risk factors for stress, after determining the average value for a specific given question. At the same time, the analysis carried out allows us to follow the dynamics of changes in the impact of identified hazardous psychosocial stress risk factors on the experience of stress after the application of preventive and protective measures. However, similar approaches are criticized due to the presence of subjective biases in the answers and the problem of identifying causal relationships (47).

There is also a need to formulate questions that would allow experts to identify the relevant relationships between significant hazardous psychosocial stress risk factors, stress risk and the consequences of stress at work with the development of certain employee diseases that develop under the influence of stress experienced by the employee. It is assumed that both the form of the questionnaire and the questions themselves can be changed by adding questions that would determine the impact of the specifics of work in the organization or the level of satisfaction of employees, or their involvement in improving the management system, quality and labour productivity (47). This possibility is foreseen in works where the authors clarify and adjust known questionnaires for specific enterprises (19, 48).

In order to reduce the influence of biases in the formation of scales or questions, it is suggested to involve a group of experts who will conduct a study of the workplace and select relevant questions that will most closely correspond to the existing influences on aspects of organizational work, social factors, equipment, the working environment, and it is also recommended to detect the level of deterioration health, which is recorded in the medical records of employees (49, 50). In this study, there was no such check, since the survey was conducted voluntarily, and the received information about the state of health was recorded only if the participants of the survey wished to disclose it. As for the correlation between the parameters of the scale for determining significant dangerous psychosocial stress risk factors, they generally coincide with the expectations in the literature (48, 51).

In the presented process of determining significant hazardous psychosocial stress risk factors, it is proposed to divide the answers between male and female employees, which will allow a better understanding of the reasons that cause the experience of professional stress. For example, having set the average answer to 1.85 points for question *НF_3-9_*, according to the proposed scale, we can see that the hazardous psychosocial risk factor of stress, which refers to the employee’s experience of ensuring an appropriate level of safety at the workplace, is insignificant, while for women, on the contrary, this question needs detailed consideration, since the level of their answers is 2.3 points. This approach is of immense importance in maintaining the psychological stability of both an individual employee and the team as a whole. Because it affects the psychological climate in the work team and allows managers to find an individual approach to each member of the team, building interpersonal relationships and communicative activity (52, 53).

Further research is expected to refine the scale to determine the significance of the impact of hazardous psychosocial stress risk factors based on a comparison of changes in the level of health when performing professional activities under the influence of certain groups of hazardous stress risk factors. The authors plan to conduct research in post-war conditions and compare it with the results of those held in wartime conditions. Consider the division into other groups of workers, in particular by age. It is necessary to develop a process of assessing the psychosocial safety of employees in the units of the enterprise, to determine where there is a low level of mental health of employees, and to carry out preventive and protective measures to reduce the impact of hazardous psychosocial risk factors of stress on employees.

## 5 Conclusion

The developed PSS assessment system will enable users to systematically observe the state of mental health of employees, the psychological climate in the organization, respond in a timely manner to expected problems and develop corrective actions to normalize the situation.

The suggested process of managing significant hazardous psychosocial risks consists of ten steps and differs from the known procedure of identifying significant hazardous psychosocial factors on a Likert scale, considering the answers of women and men.

It was found that women pay more attention to challenges (psychosocial hazardous factors) that are associated with the organization of work (uncertainty at work, lack of breaks due to workload) and social problems (disrespect, disrespect and inattention to employees, unreasonable behavior towards you by leadership); for men, a significant group of hazardous psychosocial factors is–equipment, working environment, hazardous tasks (fear of performing hazardous work; work is associated with a significant risk to life).

## Data Availability

The original contributions presented in the study are included in the article/supplementary material, further inquiries can be directed to the corresponding author.
